# Oxidative damage diminishes mitochondrial DNA polymerase replication fidelity

**DOI:** 10.1093/nar/gkz1018

**Published:** 2019-12-04

**Authors:** Andrew P Anderson, Xuemei Luo, William Russell, Y Whitney Yin

**Affiliations:** 1 Quantitative and Computational Biosciences, Baylor College of Medicine, Houston, TX 77030, USA; 2 Sealy Center for Structural Biology, University of Texas Medical Branch, Galveston, TX 77550, USA; 3 Department of Biochemistry and Molecular Biology, University of Texas Medical Branch, Galveston, TX 77550, USA; 4 Department of Pharmacology and Toxicology, University of Texas Medical Branch, Galveston, TX 77550, USA

## Abstract

Mitochondrial DNA (mtDNA) resides in a high ROS environment and suffers more mutations than its nuclear counterpart. Increasing evidence suggests that mtDNA mutations are not the results of direct oxidative damage, rather are caused, at least in part, by DNA replication errors. To understand how the mtDNA replicase, Pol γ, can give rise to elevated mutations, we studied the effect of oxidation of Pol γ on replication errors. Pol γ is a high fidelity polymerase with polymerase (*pol*) and proofreading exonuclease (*exo*) activities. We show that Pol γ *exo* domain is far more sensitive to oxidation than *pol*; under oxidative conditions, exonuclease activity therefore declines more rapidly than polymerase. The oxidized Pol γ becomes editing-deficient, displaying a 20-fold elevated mutations than the unoxidized enzyme. Mass spectrometry analysis reveals that Pol γ *exo* domain is a hotspot for oxidation. The oxidized *exo* residues increase the net negative charge around the active site that should reduce the affinity to mismatched primer/template DNA. Our results suggest that the oxidative stress induced high mutation frequency on mtDNA can be indirectly caused by oxidation of the mitochondrial replicase.

## INTRODUCTION

Human mtDNA codes for two rRNAs, and 22 tRNAs necessary for the translation of 13 genes essential to the oxidative phosphorylation electron transfer process (1). Integrity of mtDNA is vital to cellular functions. It is estimated that mtDNA suffers ten-fold more point mutations than its nuclear counterpart (2,3). Accumulated somatic mutations on mtDNA cause organelle and cellular dysfunction, and have been implicated in ageing, cancer and neurodegeneration (4–6).

The mechanism underlying the accumulation of mtDNA mutations is not well understood. MtDNA is located in an environment high in reactive oxygen species (ROS) [9], which are generated endogenously from the electron transport chain and metabolic redox reactions (7); the high mutation rate was therefore thought to be a product of direct oxidation of mtDNA. Because the most common DNA oxidation product is 8-oxoguanine (8oxoG), which promotes DNA polymerase to misincorporate dATP, a 30,000-fold increase in G:C to T:A transversions at the oxidized G position is expected (8). Additionally, oxidized nucleotide, 8oxodGTP can complete with dTTP and promotes A:T to C:G transversion (9), which would further increase transervation mutations. Nonetheless, mtDNA transversion is reported to be much less than transition mutations, at a ratio of 1:9 (10–12), indicating that 8oxoG either occurs at lower frequency than random mutations on mtDNA or is rapidly repaired.

In human mitochondria, 8oxoG is primarily removed by base excision repair, where the oxidized guanine is excised by 8oxoG glycosylase (OGG1) (13), and 8oxo-dGTP is eliminated by Mut homolog (MTH1) (14,15). However, loss-of-function mutations in OGG1 do not significantly influence the mtDNA mutation rate (11); Although *ogg1* or *ogg1/mth1* knock-out mice increased the levels of 8-oxoguanine in mtDNA, the overall mtDNA mutation frequency is not significantly increased (16,17). These studies suggest that direct oxidative damage or its repair is not the primary cause of mtDNA mutations, rather that it likely arises from replication errors.

Human mtDNA is replicated by DNA polymerase gamma (Pol γ), minimally together with Twinkle helicase and single-stranded DNA-binding protein (SSB) (18). Human Pol γ is a heterotrimer consisting of a catalytic subunit Pol γA and a dimeric accessory subunit Pol γB. The Pol γA subunit contains at least two active sites: a polymerase (*pol*) site for template-dependent DNA synthesis, and a 3′-5′ exonuclease (*exo*) site for proofreading (19). Pol γB subunit has no independent enzymatic activity but regulates both the *pol* and *exo* activities of the holoenzyme (20). Exonuclease activity is critical to maintain high fidelity during DNA replication (21,22). Transgenic mice with overexpressed exonuclease-deficient Pol γ in cardiac tissue rapidly accumulated mtDNA mutations up to 23-fold more than wild-type and many developed cardiomyopathy (23). Furthermore, mice carrying exonuclease-deficient Pol γ (D257A) displayed elevated mtDNA mutations; the animals exhibited a mutator phenotype and suffered from premature ageing (24). These studies established a link between increased replication errors on mtDNA and degenerative symptoms.

Because the mitochondrial replication machinery also exists in an ROS-rich environment, it is likely that ROS-induced oxidative damage to proteins of the mitochondrial replication machinery might contribute to replication errors in mtDNA. Indeed, oxidized bacteriophage T7 DNA polymerase (T7DNAP) displayed greater reduction in exonuclease than polymerase activity (25). Pol γA shares structural and functional homology with T7 DNAP, and is more sensitive to oxidation than human Pol α and Pol β (26,27). These observations raise the question of whether oxidized Pol γ could negatively impact mtDNA integrity.

Here we report studies on oxidation-induced activity changes in Pol γ. Oxidized Pol γ exhibits a 20-fold reduction in exonuclease activity while polymerase activity is relatively unchanged, suggesting upon oxidation, the high fidelity Pol γ is converted into an editing-deficient polymerase. Mass spectrometry analyses further reveal that the Pol γ exonuclease active site is a hotspot for oxidation. Our results thus indicate that Pol γ could be a major contributor to elevated mtDNA mutations under conditions of oxidative stress.

## MATERIALS AND METHODS

### Materials

Synthetic oligonucleotides (Table 1) were purchased from Integrated DNA Technologies (Coralville, Iowa), dNTPs and restriction enzymes were obtained from New England BioLabs (Ipswich, MA, USA). S-Trap Columns were obtained from PROTIFI (Huntington, NY, USA)

**Table 1.** Oligonucleotide sequences

Oligonucleotide Name	Sequences
M13-26-primer (26 nt)	5′-GGATTATTTACATTGGCAGATTCACC
MM-p (26 nt)	5′-GGATTATTTACATTGGCAGATTCAAT
MM-t (40 nt)	5′-AATCTAGTCCCAAGCTTGAATCTGCCAATGTAAATAATCC
MM-p/t duplex (26/40 nt)	3′-CCTAATAAATGTAACCGTCTAAGTT**C**GAACCCTGATCTAA
	5′-GGATTATTTACATTGGCAGATTCAA**T**
Buried Mismatch 40 bp (B40 bp)	3′-CCTAATAAATGTAACCGTCTAAGTT**C**GAACCCTGATCTAA
	5′-GGATTATTTACATTGGCAGATTCAA**T**CTTGGGACTAGATT
Excised Mismatch 40 bp (E40 bp)	3′-CCTAATAAATGTAACCGTCTAAGTT**C**GAACCCTGATCTAA
	5′-GGATTATTTACATTGGCAGATTCAA**G**CTTGGGACTAGATT
	HindIII site

### Pol γ and DNA preparation

Pol γ subunits Pol γA and Pol γB were expressed, respectively, in sf9 insect cells and BL21(DE3)-RIL bacteria and purified as previously described (27). Briefly, the proteins were purified using Ni-NTA, RESOURCE S and RESOURCE Q affinity chromatography, followed by gel filtration on a Superdex 200 column.

M13mp18 DNA was purified from infected IJ338 cells (K-12 *ara thi* Δ(*pro-lac*) *supD*-Tn*10*/F'*traD36 proA^+^B^+^ lacI^Q^* l*acZΔM15*, a kind gift from Dr. Ian Molineux). A single colony of IJ338 was isolated and grown in LB with tetracycline to mid-log phase at 37°C. The culture was brought to 1 mM IPTG, 400 μg/ml X-gal, and a dilution series of phage was added, and then mixed into soft agar before being poured onto agar plates. After overnight growth at 37°C, a single blue plaque was extracted and added to mid-log phase IJ338, then incubated for 30 min at 37°C. This solution was then used to inoculate a large volume of LB, which was grown overnight at 37°C. The overnight culture was centrifuged at 10,000 RCF for 10 min, and the supernatant was brought to 3% PEG 8000, 0.5 M NaCl and left overnight at 4°C to precipitate the phage. After centrifugation at 5000 RCF for 10 min at 4°C the phage pellet was resuspended in 10 mM Tris pH 7.6, 10 mM NaCl and the single-stranded M13 mp18 circular DNA was purified by phenol-extraction.

Duplex DNAs were annealed by heating complementary DNA strands in 150 mM NaCl and 20 mM HEPES (pH 7.5) to 95°C for 5 min and slow cooling overnight to 20°C. 5′-^32^P oligonucleotide labeling used γ-^32^P-ATP and T4 polynucleotide kinase as per the manufacturer (New England Biolab) instructions.

### Oxidation of Pol γ

Pol γ (5 μM) was incubated with 50, 100, 250, 500 μM or 1 mM hydrogen peroxide (H_2_O_2_) at 37°C for 1 h. Oxidized Pol γ integrity was analyzed by dynamic light scattering (DLS) using a Malven Zetasizer μV apparatus. Briefly, 2.75 μM untreated and 1 mM H_2_O_2_-treated Pol γ were measured at 830 nm at 20°C in solution containing 20 mM HEPES (pH 7.5), 140 mM KCl and 5% glycerol. Data were processed using Zetasizer 7.11 software.

### Exonuclease activity assays

Reactions were carried out in RX buffer (10 mM HEPES pH 7.5, 10% glycerol, 140 mM KCl and 1 mM EDTA). 200 nM Pol γ was pre-incubated with 500 nM 5′-^32^P-26 nt DNA (M13-26-primer in Table 1) for 5 min at 37°C. Reactions were initiated by the addition of 10 mM MgCl_2_, and quenched at the indicated times by addition of four reaction volumes of Q buffer (80% formamide, 1% SDS, 25 mM EDTA, pH 8.0) and heated at 95°C for 5 min. Reaction products were resolved by electrophoresis on a 20% denaturing (7M urea) polyacrylamide gel and quantified on a GE Typhoon 9500 at 100 μm using ImageQuant 7.1. The apparent rate of substrate digestion was estimated by fitting the time-dependent fraction of substrate reduction from the initial rate for untreated and H_2_O_2_ oxidized Pol γ.

### Polymerase activity assay

50 nM Pol γ was pre-incubated with 100 nM M13 mp18 ssDNA annealed with the 5′-^32^P-26 nt primer (M13-26nt-primer, Table 1) in RX buffer for 5 min at 37°C. Reactions were initiated by addition of 400 μM each dNTPs and 10 mM MgCl_2_ and quenched with Q buffer at the indicated times. Reaction products were heated at 95°C and then resolved on an 8% denaturing (7 M urea) polyacrylamide gel and quantified by ImageQuant 7.1

### Mismatch primer DNA excision and extension

Mismatch excision assays were conducted with 100 nM Pol γ pre-incubated with 800 nM 26/40nt DNA containing a single mismatch at the primer 3′-end (MM p/t, Table 1). Excision of the mismatch was monitored as described above in *Exonuclease activity assay* on ssDNA.

Mismatch primer extension was carried out on the same 26/40 nt MM p/t DNA in the presences of 100 μM dNTP, 10 mM MgCl_2_ for 30 min. The reactions were quenched as designated time by addition of buffer Q and heating to 95°C for 5 min. To distinguish Pol γ mismatch removal in the coupled excision and extension reactions, duplicated samples were prepared where HindIII was added following the mismatch p/t synthesis. Reaction products were analyzed on a 20% denaturing acrylamide gel and quantified by ImageQuant 7.1.

### Mass spectrometry

#### Sample preparation

Samples were proteolyzed by either trypsin or combination of trypsin and GluC. Prior to protease digestion, 110 μl untreated or oxidized Pol γ heterotrimer (1μM) were denatured in 5% SDS at room temperature overnight, then mixed with 4μl iodoacetemide (0.5M) for 40 minutes to alkylate the cysteine residues while being protected from light. 10 μl phosphoric acid (12%) was added to the protein solutions. The proteins were diluted with 650 μl S-Trap Buffer (100 mM triethylammonium bicarbonate (TEAB, pH 8.0) and 90% methanol), and applied to S-Trap Mini Spin Columns. The columns were centrifuged at 1000 RCF for 2 min and washed twice with 150 μl S-Trap buffer.

For trypsin only digestion, trypsin in 100 mM TEAB (pH 8.0) was added to the S-Trap column at a 1:10 weight to weight ratio of Pol γ and digested at 37°C overnight. The digested peptides were eluted by centrifugation at 1000 g for 2 min sequentially with the following solution: 40 μl TEAB (pH 8.0), 40 μl 0.2% aqueous formic acid, 35 μl 50% acetonitrile and 0.2% formic acid, 80% acetonitrile and 0.2% formic acid. The elutants were combined and dried in a SpeedVac vacuum concentrator and reconstituted in 2% acetonitrile and 0.1% formic acid.

For trypsin and GluC digestions, the trypsin overnight digestion was eluted with 40 μl 100 mM TEAB (pH 8.0) solution, to which GluC was added at a 1:10 weight to weight ratio of Pol γ. The protease solution was loaded onto a S-Trap Column and digested at 47 °C for 2 h. The digested peptide was eluted using the procedure described above for final elution of peptides digested by trypsin alone.

#### Data acquisition and analyses

Nano-LC/MS/MS was performed on a Thermo Scientific Orbitrap Fusion system, coupled with a Dionex Ultimat 3000 nano HPLC and auto sampler with 40-well standard trays. Samples were injected onto a C18 PepMap100 trap column (300 μm × 5 mm) followed by a C18 reverse-phase nano LC column (75 μm × 25 cm, Thermo Fisher Scientific) at flow rate of 400 nl/min with an acetonitrile gradient (0–99.9% in 0.1% formic acid) for 60 min at 50°C. The eluted peptides were sprayed through a +2.2 kV charged 10 ± 1 μm emitter tip (PicoTip Emitter, New Objective) into the mass spectrometer. Fourier transform mass spectrometry mode was enabled for acquisition of precursor ions, with resolution set to 120 000, and Ion Trap mode was used for subsequent MS/MS via higher-energy collisional dissociation at top speed every 3 s.

Proteome Discoverer 1.4 was used for protein post-translational modification identification and peak detection. The UniProt human database was used to analyze raw data. Carbamidomethyl cysteine, deaminated asparagine and glutamine as well as other amino acids modifications were treated as dynamic modifications. Two missed protease cleavages were allowed and the precursor mass tolerance was set at 10 ppm. MS/MS fragment mass tolerance was set at 0.6 Da, and peptides with +2, +3 or +4 charges were considered. Only peptides with a false discovery rate <1% were considered.

For a given residue *r*, its oxidation was defined as in Equation 1: The fraction oxidized (*f_r_*) is the sum of the peptide (*P*) peak heights (*h_P_*) that contain the oxidized (*ox*) residue divided by the sum of the heights of any form of the peptide. The relative residue oxidation (*F_r_*) is *f_r_* normalized against the average oxidation fraction (*f’*).

(1) $$\begin{equation*}{{{f}}_{{r}}} = \frac{\sum ({Hp\, of\, peptides\, containing\, r}_{ox})}{\sum (Hp\, of\, all\, peptides\, containing\, r)}\ ,\,{{{F}}_{{r}}} = \frac{{{{{f}}_{{r}}}}}{{{{f^{\prime}}}}}\end{equation*}$$

Oxidized arginines may prevent trypsin digestion, thus reduces the peptide detection in oxidized regions. Furthermore, we set the threshold for oxidation high to reduce false positives. This stringent search strategy could increase false negatives but should decrease false positives, thus likely underestimates oxidized residues.

## RESULTS

### Oxidation reduces Pol γ exonuclease activity

**Figure 1.**
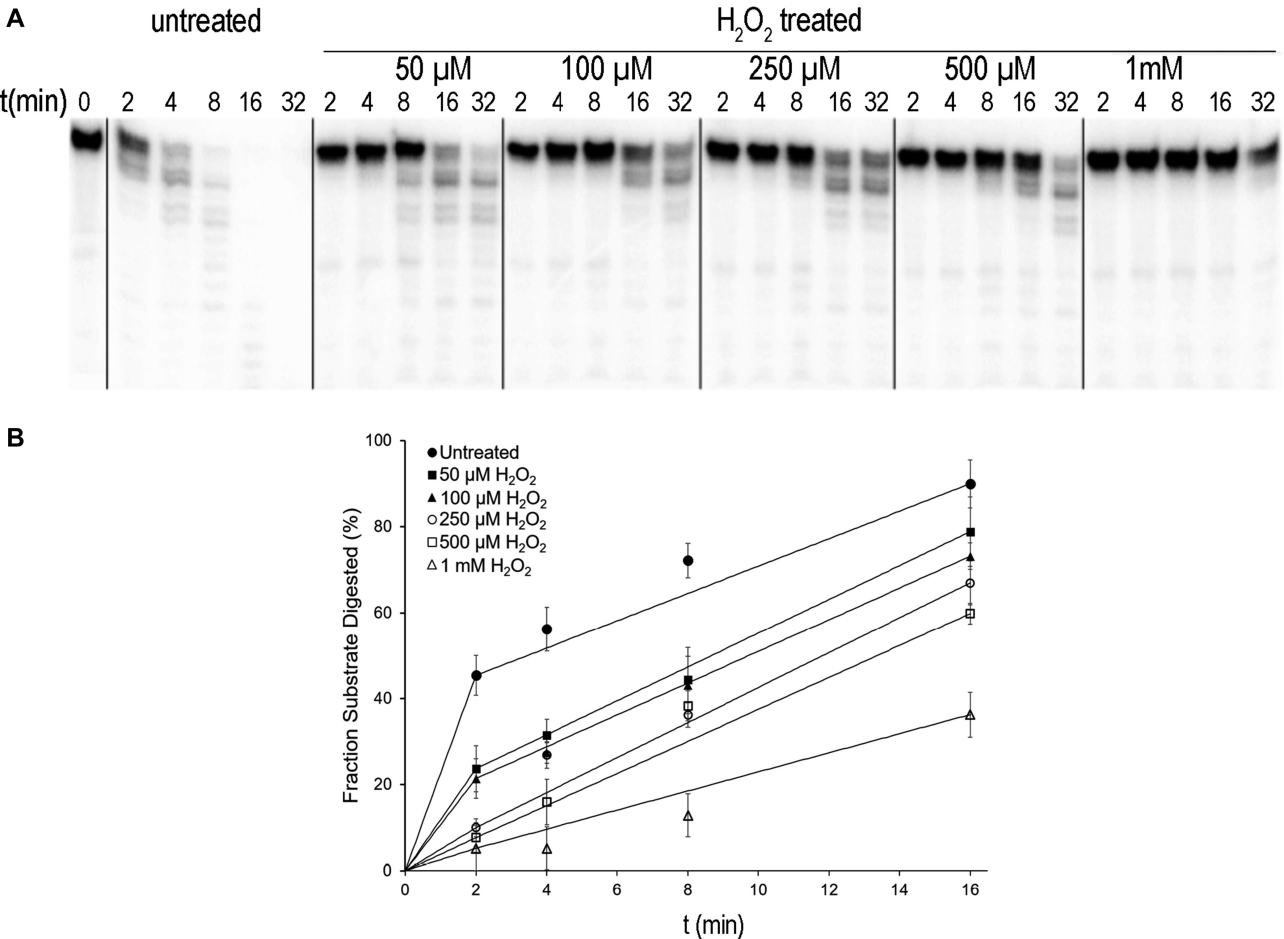
Oxidation decreases Pol γ exo activity. (**A**) the time-dependent exonuclease activity on single-stranded DNA. (**B**) Quantification of digested substrate from each sample normalized by the total accounts within the lane. The means and standard errors are calculated from three independent experiments.

The 3′→5′ exonuclease activity of untreated and oxidized Pol γ was assayed using a 5′-^32^P-26 nt single-stranded DNA (M13-26-primer in Table 1). The nuclease activity was measured by quantifying the remaining substrate normalized by the total density within the lane, and then fitted to single exponential model to determine the apparent reaction rates (Figure 1A).

The rate of ssDNA degradation by untreated Pol γ was 0.25 s^−1^, those for oxidized Pol γ treated with 50 μM, 100 μM, 250 μM, 500 μM and 1 mM hydrogen peroxide were 0.19, 0.16, 0.13, 0.11 to 0.03 s^−1^, respectively (Figure 1B). Even at the lowest H_2_O_2_ treatment concentration the polymerase already shows an 24% reduction in nucleolytic rate. The maximally oxidized Pol γ showed a greater than 8-fold reduction in nuclease excision rate relative to the untreated enzyme.

### Oxidation does not damage Pol γ physically

To analyze whether oxidation-induced Pol γ activity changes was caused by its physical damage, we compared molecular dimension of untreated and oxidized Pol γ by dynamic light scattering to detect aggregation or protein unfolding. The radius of the enzyme oxidized with 1mM H_2_O_2_ was 7.7 nm compared to 7.4 nm for the untreated enzyme; both forms were also monodispersed (Supplementary Figure S1A). No crosslinking of Pol γ due to H_2_O_2_ treatment was detected by SDS PAGE (Supplementary Figure S1B), we thus conclude that oxidative treatment with H_2_O_2_ does not cause significant physical alterations to Pol γ.

### Oxidized Pol γ maintains normal polymerization activity

**Figure 2.**
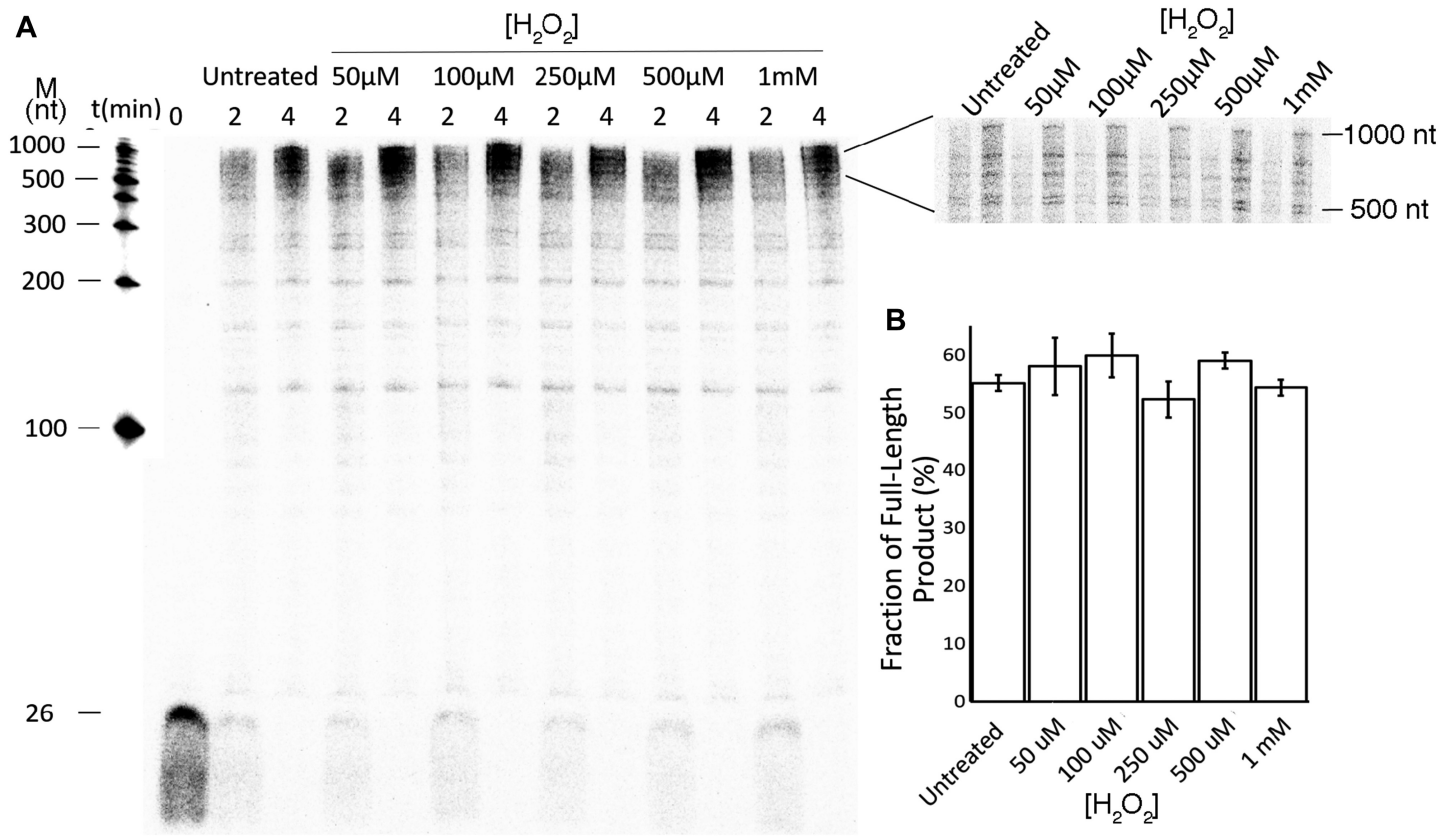
Oxidized Pol γ pol activity. (**A**) DNA synthesis activity of untreated and treated Pol γ measured on a singly primed M13mp18 ssDNA, (**B**) Quantification the fraction of full-length products that sums the product between 500 and 1000 nt then normalized by the total counts within the lane. Data were averaged from three independent reactions.

DNA synthesis activity of oxidized Pol γ were carried out on a singly primed M13 mp18 ssDNA by a 5′-^32^P-26nt primer (M13-26-prime, Table 1). Due to the location of the primer to a long hairpin at the M13 replication origin, the maximum extended product is ∼1000 nt. The untreated and oxidized Pol γ exhibited similar activities. Quantification of time-dependent full-length (sum of 500–1000 nt) products showed that polymerase oxidized with 50 μM, 100 μM, 250 μM, 500 μM and 1 mM H_2_O_2_ all produced comparable products in length and quantity to the untreated enzyme (Figure 2). As the primer is end-labeled, the length and quantify of DNA correlate with processivity and synthesis rate. The results suggest that oxidation has differential effects on Pol γ *pol* and *exo* activities, unlike *exo* activity, the *pol* activity is much less sensitive to oxidation.

### Synthesis at mismatched DNA

**Figure 3.**
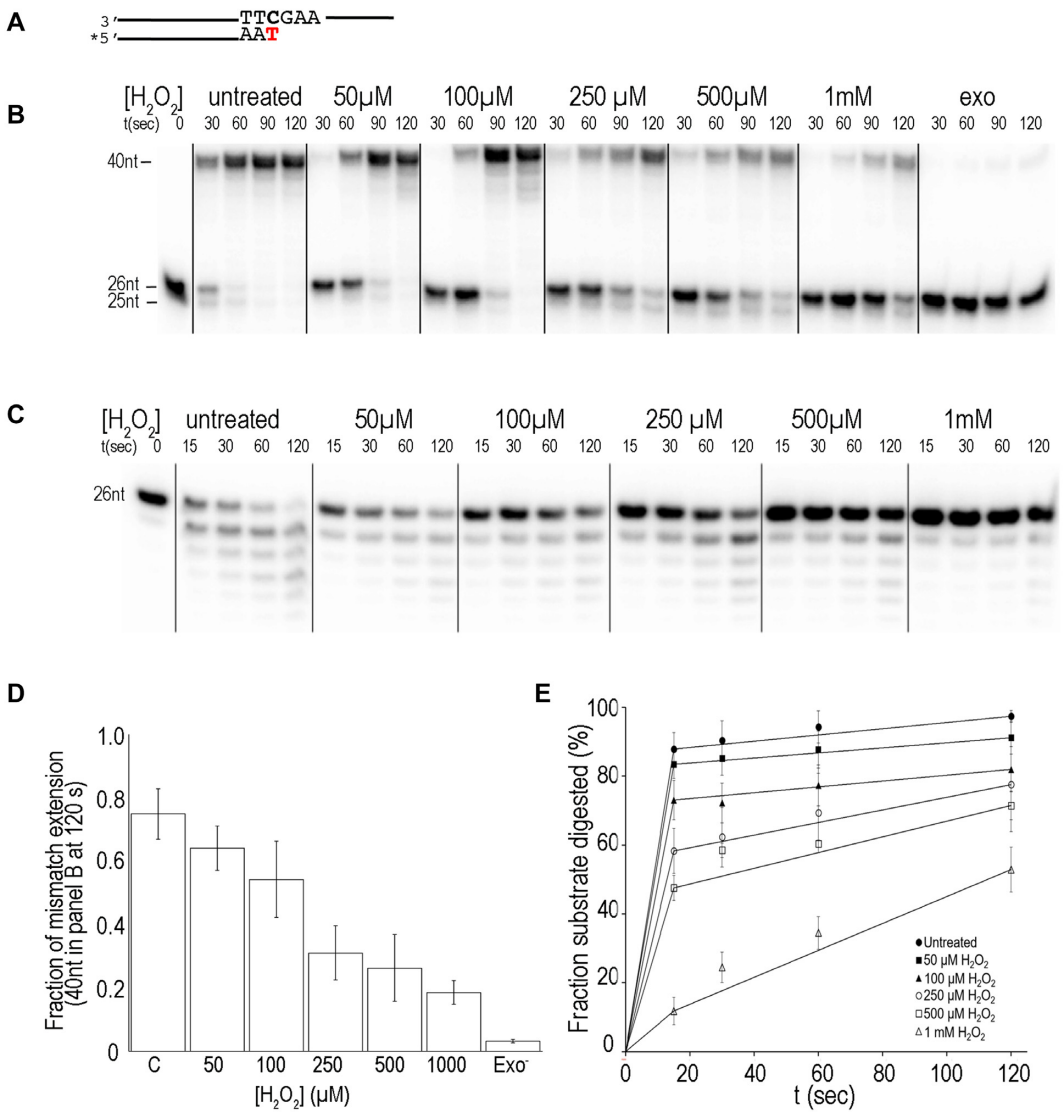
Effects of oxidation on mismatch extension and excision. (**A**) The schematic of the substrate with a 1-nt mismatch at the 3′-end primer used in primer extension and excision assays. The asterisk indicates 5′-^32^P labeling. (**B**) Time-dependent mismatched primer extension by untreated, treated and exo-deficient Pol γ. (**C**) Mismatched primer excision by untreated, H_2_O_2_ treated, and exo-deficient Pol γ. (**D**) Quantification of fraction of mismatch primer extension to full-length at 120 s from data presented in (B). (**E**) Quantification mismatched primer in panel (C). The mean and standard deviation were determined from three independent experiments.

The above results suggest that oxidation maintains Pol γ DNA synthesis but reduces proofreading, thus the oxidized Pol γ should become more error-prone relative to the untreated polymerase. To test the hypothesis, we measured oxidized Pol γ’s ability to synthesize on the 26/40 nt dsDNA where the primer 3′-end residue cytosine is a mismatch with the templating T (Figure 3A), which mimics the product of erroneous nucleotide incorporation. The mismatched DNA therefore can assay Pol γ’s nucleolysis and polymerase activities simultaneously.

Synthesis of the mismatched DNA was assayed with untreated, oxidized, and exo-deficient Pol γ (exo^−^, containing D198A/E200A substitutions). Pol γ exo^−^ retains wild-type *pol* activity but ∼1% of *exo* activity (28), therefore should only perform error-prone DNA synthesis by burying the mismatch. Within 60 s, untreated Pol γ completely extended the mismatched primer to full-length 40 nt, but Pol γ exo^−^ produced less than 1% of 40 nt, indicating extension of the 3′-mismatch primer is very slow. The oxidized Pol γ displayed H_2_O_2_ concentration-dependent reduction of full-length DNA. The rate of full-length synthesis for the most oxidized Pol γ (1 mM H_2_O_2_) is ∼10% of the untreated enzyme. Notably, relative to untreated Pol γ, the excised product 25 nt appeared later in time for the oxidized samples, and the delay is correlated with H_2_O_2_ concentration. The delayed appearance of the 25 nt DNA is consistent with reduced exonuclease activity in the oxidized polymerase.

We next tested oxidized Pol γ proofreading activity on the same mismatched 26/40 nt duplex. While the untreated Pol γ excised 50% of the mismatch nucleotide from the primer in 15 s, resulting apparent rate 1.66 s^−1^ for the mismatch removal. Smaller products appeared after longer reaction times, suggesting correctly base-paired primer being excised, but at slower rate than the unpaired. Comparison to the quantity of 25 nt made by untreated enzyme, the least oxidized Pol γ (50 μM H_2_O_2_) is 2.5-fold less, and the most oxidized Pol γ (1 mM H_2_O_2_) is 11-fold less efficient in mismatch removal (Figure 3C). The results suggest slow extension of mismatched primer by oxidized and exo^−^ Pol γ is, in part, caused by reduced mismatch removal, and mismatch-burying extension is slower than the combined rates of error removal and error-free extension.

### Fidelity of the oxidized Pol γ

**Figure 4.**
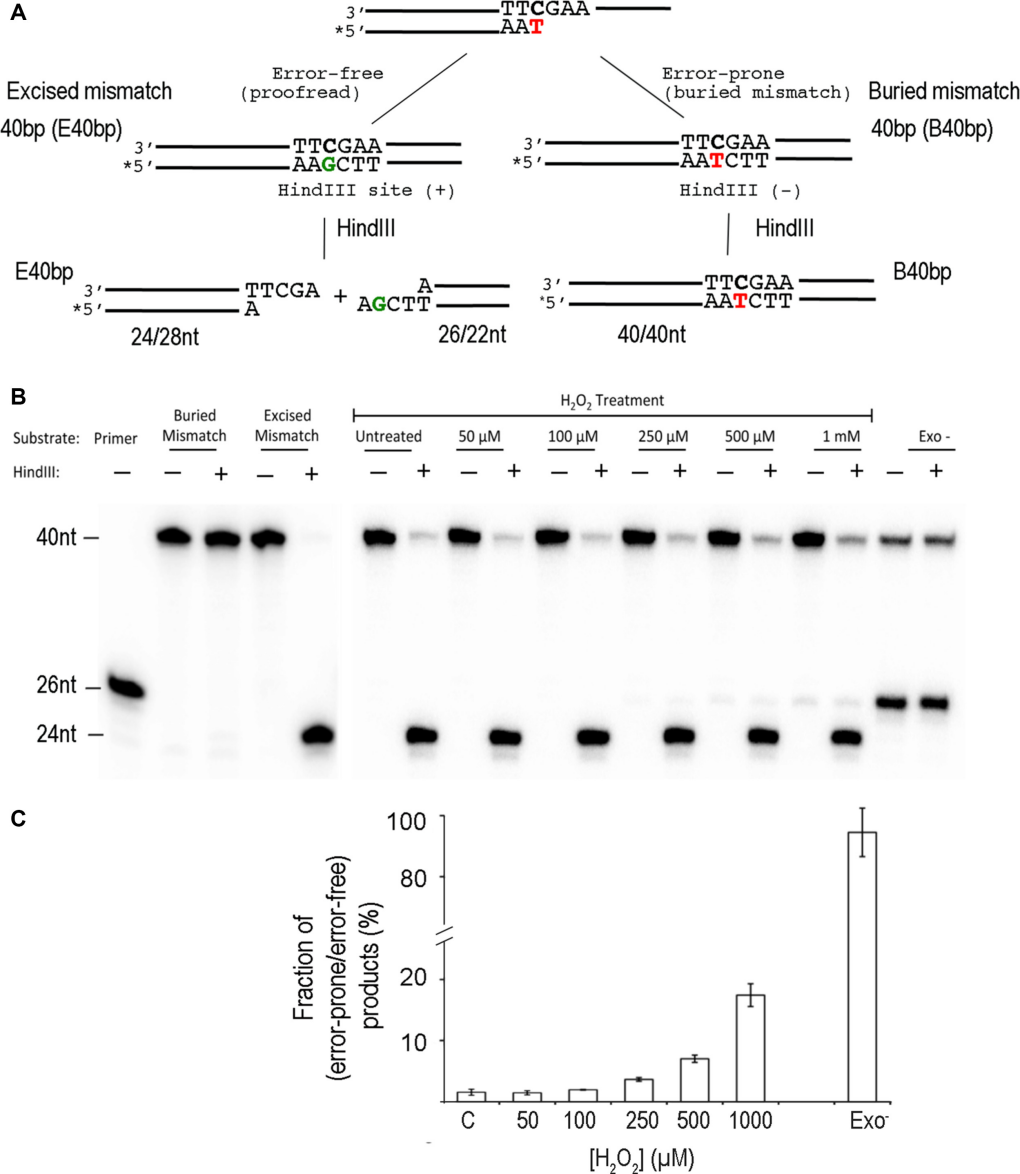
Effect of oxidation on polymerase replication fidelity. (**A**) The scheme for distinguishing error-free and error-prone DNA synthesis on the designed mismatched primer/template DNA. The asterisk indicates 5′-^32^P labeling. (**B**) Products of mismatched DNA extension and digestion by HindIII by Pol γ treated with H_2_O_2_ and the *exo*-deficient variant. (**C**) Quantification of the undigested band divided by the sum of all density within the lane.

The above results suggest that the extension from the mismatched primer is a mixture of mismatch containing and corrected products. We designed the 26/40 nt mismatch dsDNA that allows for distinguishing error-prone from error-free synthesis by including a HindIII site in the error-free full-length product. The error-free product (E40 bp, Table 1) from Pol γ excision of the mismatch will constitute a HindIII site and will be cleaved by the restriction enzyme into two fragments of 24/28 nt and 22 nt/26 nt, whereas the full-length product buried the mismatch (B40 bp, Table 1) will be resistant to HindIII digestion and remains as an intact 40 bp DNA in the presence of HindIII (Figure 4A). The ratio of HindIII undigested to digested 40 bp DNA gives rise ratio of error-prone to error-free synthesis products.

Mismatch extension assays were carried out for 30 min to allow oxidized Pol γ to produce substantial 40 bp DNA for analyses, and the dNTP concentration is kept low (100 μM) to reduce misincorporation. While the untreated Pol γ extended 82% of the primer to full-length (40 nt) product in 60 s (calculated from data in Figure 3B), Pol γ exo^−^ generated only 30% of the full-length product in 30 min (Figure 4B), suggesting the polymerase generated 82-fold more full-length products from the correctly base paired primer than mismatch-burying synthesis at a unit time. The error-prone/error-free product ratio for the untreated Pol γ is <0.9%, whereas the ratio for Pol γ exo^−^ is 94%, as majority of the 40 bp DNA were undigested. The date show that the *exo* proofreading activity can correct >99% of misincorporated nucleotide, potentially reducing replication error by ∼100-fold, which are in good agreement with previously reported exonuclease activity on DNA replication fidelity by Pol γ and other high fidelity polymerases (28–31).

Pol γ error-free DNA synthesis declines with increased H_2_O_2_ concentration. Within reported physiological H_2_O_2_ concentration (50–200 μM), the error-rate of oxidized Pol γ increased 2–3-fold relate to the untreated. The ratio error-prone/error-free product for Pol γ oxidized at 1 mM H_2_O_2_ is merely 18%, which is 20-fold higher than the untreated Pol γ and only 5-fold lower than the catalytic residue double mutant exo-deficient variant (Figure 4C). These results suggest that Pol γ can significantly increase mutations during DNA replication under oxidative stress. Furthermore, as the oxidation does not affect *pol* activity on correctly base paired DNA, the overall reduced synthesis on mismatch containing DNA by oxidized Pol γ is caused by combination of slow rates of error correction and mismatch burying DNA synthesis.

### Identification of oxidation hotspots

To reveal the chemical basis for oxidation-induced Pol γ functional changes, we used liquid chromatography with tandem mass spectrometry (LC–MS/MS) to identify residues oxidized by H_2_O_2_. Analyses were carried out in replicas of untreated Pol γ and treated with 500 μM H_2_O_2_ or 1 mM H_2_O_2_. Pol γ is a holoenzyme comprised of a catalytic subunit Pol γA and an accessory subunit Pol γB. The Pol γA used in this study contains 1204 amino acids which lacks the N-terminal 35 amino acids mitochondrial localization sequence; its exonuclease (*exo*) domain is located within residues 170–440, and polymerase (*pol*) domain in residue 441–475 and 784–1239. Among five LC–MS/MS experiments (two untreated and three oxidized samples), the overall recovered peptides are within 70–90% of the total residues (Table 2). Only residues detected in both untreated and treated samples, i.e. 926 amino acid in Pol γA and 331 in Pol γB, were used for further analyses.

**Table 2.** MS analyses coverage

	Trial 1		Trial 2		
H_2_O_2_ treatment	untreated	1 mM	untreated	0.5 mM	1 mM
Coverage of Pol γA Subunit	0.77	0.69	0.83	0.80	0.84
Coverage of Pol γB Subunit	0.75	0.85	0.94	0.93	0.89

**Figure 5.**
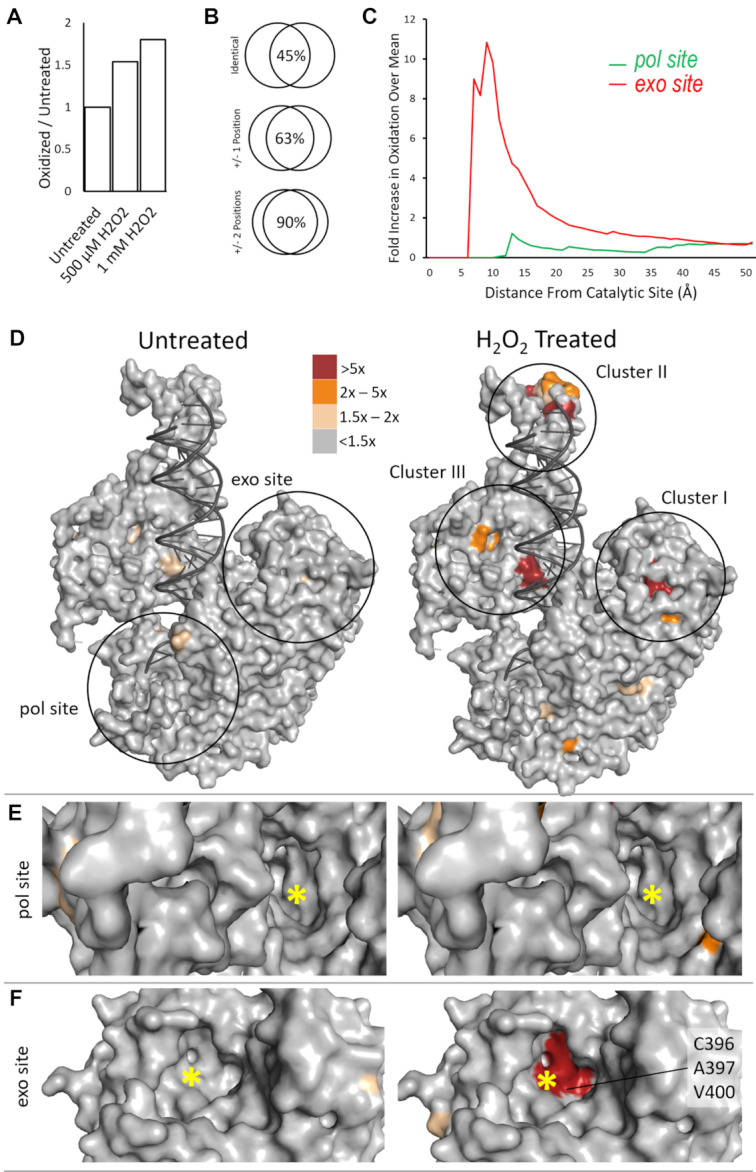
Structural illustration of oxidized Pol γ residues. (**A**) The overall fraction of oxidation (*F_r_*, Equation 1) for 500 μM and 1 mM H_2_O_2_ treated Pol γ, relative to the untreated. (**B**) Consistency of oxidized residues from three different oxidation experiments (**C**) The average oxidation *Fr* of *pol* (red) and *exo* (green) active sites. (**D**) Oxidized residues of untreated (left) and 1 mM H_2_O_2_ treated (right) catalytic subunit displayed on Pol γ holoenzyme ternary complex structure (PDB ID: 4ztz). Oxidation of each residue is color-coded by *F_r_*. Oxidation clusters are indicated. (**E**) Oxidation of the *pol* site. The asterisk marks the location of *pol* catalytic residues D890. (**F**) Oxidation of the *exo* site. The asterisk marks the exo catalytic residue D198.

The degree of overall oxidation increases with elevated H_2_O_2_ concentrations, displaying 1.5- and 1.9-fold increase over the untreated protein for the 0.5 and 1 mM treated polymerase, respectively (Figure 5A). Nevertheless, the amino acid locations of the most oxidized residues are highly consistent among treated proteins, regardless of H_2_O_2_ concentration. Among three different experiments, 45% detected oxidized residues are at identical locations, 63% are within ±1 and 90% are within ±2 positions from each other (Figure 5B). For example, adjacent residues Ala^266^ and Phe^267^ were detected in different experiments, which were denoted as Ala^266^/Phe^267^ (Table 3). The variation could be attributed to either partial oxidized residue assignment uncertainty in the processing software or spatially adjacent residues can all be oxidized but detected independently in different experiments. In either case, the MS method can identify oxidized amino acids with precision of ±2 positions.

**Table 3.** Top 20 most oxidized amino acid clusters in Pol γ detected by mass spectrometry

Subunit	Amino acid	Domain
A	Ser296	Exo
A	Cys396	Exo
A	Ala397	Exo
A	Val400, Trp401	Exo
A	Thr403, His404	Exo
A	Asp482	Exo
A	Asp487	Exo
A	Leu528,Leu530	Spacer
A	C533	Spacer
A	Glu535,Glu536	Spacer
A	Glu538	Spacer
A	Gln540,Gln541	Spacer
A	Ala547,Cys548	Spacer
A	Gln595	Spacer
A	His1110, Leu1111	Pol
B	Phe266,Ala267	
B	Asn282	
B	His313,Tyr315	
B	Leu347,Ala348	
B	Tyr432	

Note: the residues listed on the same line are those detected within ±2 positions from each other in separate experiments.

To detect any oxidation clusters, or ‘hotspots’, we mapped the oxidized residues on the Pol γ–DNA–dNTP ternary complex crystal structure (32). Both Pol γA and Pol γB subunits have highly oxidized amino acids. Of the top 20 most oxidized residues, 15 are located on Pol γA and five on Pol γB (Table 3). The oxidized residues on Pol γB are centered around Ala^266^/Phe^267^ and Asn^282^. As Pol γB is a homodimer, these residues could be located on either the proximal monomer at the interface with Pol γA or the distal monomer that is nonfunctional to the holoenzyme (Supplementary Figure S2). The mass spec analysis alone cannot provide definitive identification. However, Oxidation of the proximal Pol γB monomer would affect the holoenzyme formation and potentially reduce DNA synthesis processivity, whereas oxidation of the distal monomer oxidation has no or minimum effect on Pol γ processivity. As the holoenzyme DNA synthesis is unaffected, the oxidized residues are probably located on the distal monomer of Pol γB.

On the contrary, Pol γA oxidized residues are clustered around three areas that could significantly impact the polymerase function. Using *K*-means clustering method, three oxidation hotspots were revealed, which were designated as Clusters I, II and III.

**Figure 6.**
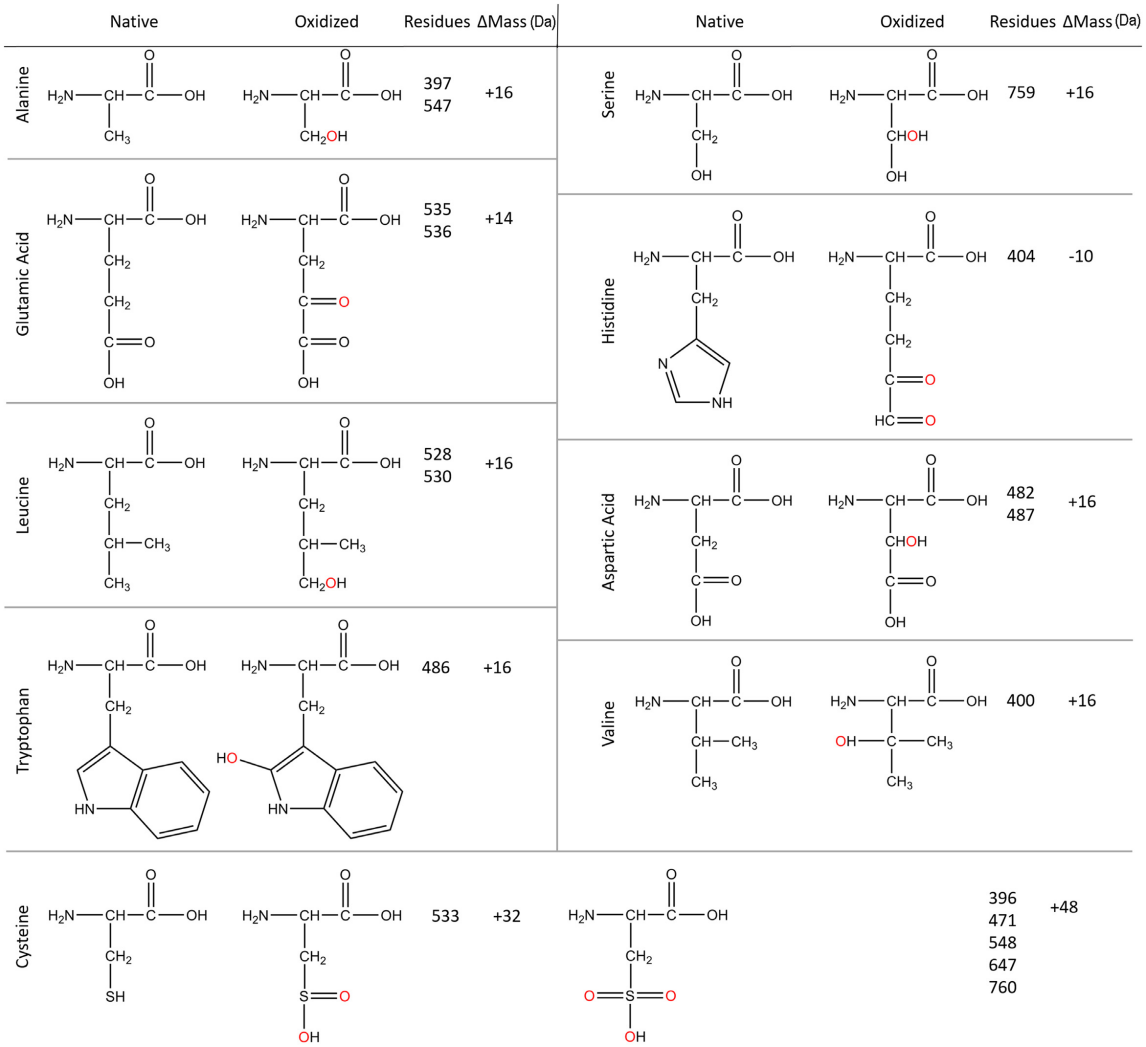
Chemical structures of detected oxidized residues.

Cluster I is located in the *exo* domain and contains 22 oxidized residues (Figure 5D). Cluster I displayed an average Relative Oxidation Fraction (*F_r_*, Equation 1) 60-fold higher than the protein mean (ƒ'). Residues Cys ^396^, Ala ^397^, Val^400^ and His^404^ are among the most oxidized residues in both 0.5 mM and 1 mM H_2_O_2_ treated samples (Table 3). Ala^397^ and Val^400^ were hydroxyl- modified, and C^396^ was converted to sulfonic acid with addition of two carbonyl oxygens to the sulfur. His^404^ lost its imidazole ring and was converted to 2-amino-5,6-dioxohexanoic acid (Supplementary Figure S3, Figure 6). Structurally, Cys^396^, Ala^397^ and Val^400^ are highly conserved among mitochondrial polymerases (33); His^404^ is conserved not only among mitochondrial DNA polymerases but also in Pol I family members. Oxidation of the histidine in T7 DNAP reduces exonuclease activity (25). Although we did not detect oxidative damage to the catalytic residues (Asp^198^ and Glu^200^) for exonuclease activity, all above oxidative modifications increase negative charges in the *exo* active site (Figure 5F), which would reduce *exo* activity by reducing its affinity to DNA substrate.

Cluster II contains 13 residues in the AID subdomain of the Spacer domain (Figure 5D). The AID subdomain (residues 511–570) (27) interacts with the accessory subunit Pol γB in the holoenzyme primarily via the L-helix and disordered loops (27). The region displayed an average *F_r_* value 80-fold higher than the ƒ’ (Table 3), specifically, residues on the L-helix Leu^528^/Leu^530^, Cys^533^, Glu ^535^/Glu^536^ and Ala^547^/Cys^548^. Apart from the Cysteine residues which were converted to sulfonic acid, the rest of them acquired an carbonyl or hydroxyl group. Hydroxyl-Ala^547^ results in A547S missense mutation (Figure 6). However, these residue side chains face away from the subunit interface and are thus less likely to alter subunit interaction.

Cluster III contains residues on the Pol γ Thumb subdomain (Figure 5D), including Cys^471^, Asp^482^/Trp^484^, Trp^486^/Asp^487^, Cys^647^, Ser^759^/Cys^760^. Residue modifications include that Cys^533^ and Cys^548^ were converted to sulfonic acid, Glu^535^ and Glu^536^ acquired a carbonyl group, the rest of the residues acquired an hydroxyl group. The *Thumb* is critical for DNA binding and transferring the primer stand between the *pol* and *exo* sites (32), oxidation of the subdomain may alter their relative activities.

Most notably, in contrast to the highly oxidized *exo* site, no significant oxidation was found in the *pol* active site (Figure 5D-F). Within 13 Å radium of the active site, the average oxidized amino acid (*F_r_*) in *exo* site is 10.8-fold above than the protein mean, whereas that of the *pol* site is merely 1.2-fold over the protein mean (Figure 5C). These results explain the different impact of oxidation on polymerase and exonuclease activities, providing a chemical basis for the editing-deficient phenotype of oxidized Pol γ.

## DISCUSSION

Mutations on mtDNA can disrupt cellular energy supply and cell cycle control, and are implicated in physiological process of aging, and pathology of neurodegeneration such as Alzheimer's and Parkinson diseases (34). A mitochondrion contains 10–1000 copies of mtDNA; reduced mtDNA copy number and/or increased mutations will have detrimental effect on cellular and organelle functions. Factors contributing to mtDNA mutations are incomplete repair of DNA, oxidative stress and replication errors.

The mitochondrial matrix houses significant quantities of reactive oxygen species (35), which are generated continuously by the oxidative phosphorylation electron transfer chain even while operating normally (7). Reactive oxygen species (ROS) include superoxide anions, hydroxyl radical and hydrogen peroxide (H_2_O_2_). A moderate increase in ROS can promote cell proliferation and differentiation. Nevertheless, under pathological conditions, such as cancer or degenerative diseases, ROS levels are severely elevated and disrupt redox homeostasis, leading to cell death (7,37). The reported H_2_O_2_ concentration varied greatly, from 50 to 200 μM (38,39), up to 1–2% of the molecular oxygen in cells (40). We chose a wide range of H_2_O_2_ concentrations in this study to explore differential oxidative propensity of Pol γ *pol* and *exo* activities.

Mitochondrial DNA replicase Pol γ is a high fidelity polymerase and contains proofreading ability to excise misincorporation in the *exo* site. Pol γ replication error frequency is 2 × 10^−6^ where the *exo* proofreading contributes 10^1^–10^2^-fold towards error reduction (28,30,41), similar to other high fidelity polymerases (29,42). Thus, exonuclease-deficient Pol γ could potentially elevate mutations by two orders of magnitude.

We show in this study that oxidation of Pol γ can increase replication errors that is positively correlated with the level of ROS. Within a relatively wide concentration range of hydrogen peroxide (50 μM–1 mM), Pol γ maintains normal polymerase activity but displays H_2_O_2_ concentration-dependent declined exonuclease activity. Under oxidative stress, the short-term effect is that Pol γ could become an editing-deficient polymerase with proofreading ability reduced up to 20-fold. The long-term effect will be the overall mtDNA synthesis declines, because oxidized Pol γ buries rather than corrects erroneously incorporated nucleotides and synthesis of burying a mismatch is much slower than extension of a correctly matched primer. Therefore, oxidation can exert dual-negative impact on Pol γ replication: increased mutations and ultimately reduced mtDNA copy number.

The high ROS contents in mitochondria undoubtedly results oxidized mtDNA and proteins. MtDNA mutations have been observed increase with age, leading to the radicals theory in ageing where free radicals directly damage DNA thus causes mutations (52,53). A weak point of the theory is that radical induced DNA damage cannot be rescued by DNA glycosylases that repair primary oxidized nucleobase, and glycosylases level does not correlate with longevity, diminishing the contribution of direction DNA oxidation. In this study, we found that oxidation reduces Pol γ proofreading ability, which reduces replication fidelity and indirectly increases mtDNA mutations. Our results are consistent with somatic mtDNA mutations are largely random and not transversion, supporting that ROS induced mtDNA mutations are mainly results from replication errors rather than direct DNA damage. It is probable that mutations in ageing and other degenerative diseases are also the consequences of Pol γ oxidation.

Pol γ proofreading deficient mutations have been well-documented in human diseases and aging. Mice carrying *exo*-deficient Pol γ (D257A) showed only 4-fold elevated mtDNA mutation but exhibited severe premature aging phenomenon (24). Mice carrying *pol*-deficient mutant Pol γ (D1135A) displayed reduced mtDNA contents and peripheral aging characteristics with inflamed wrinkled skin and hair loss (43). Our oxidation experiments subjected Pol γ to hydrogen peroxide for only one hour, which has already produced significantly reduced *exo* activity. Higher concentration of ROS or longer exposure would generate greater damage to Pol γ, rendering *exo*-deficient or completely inactive polymerase.

**Figure 7.**
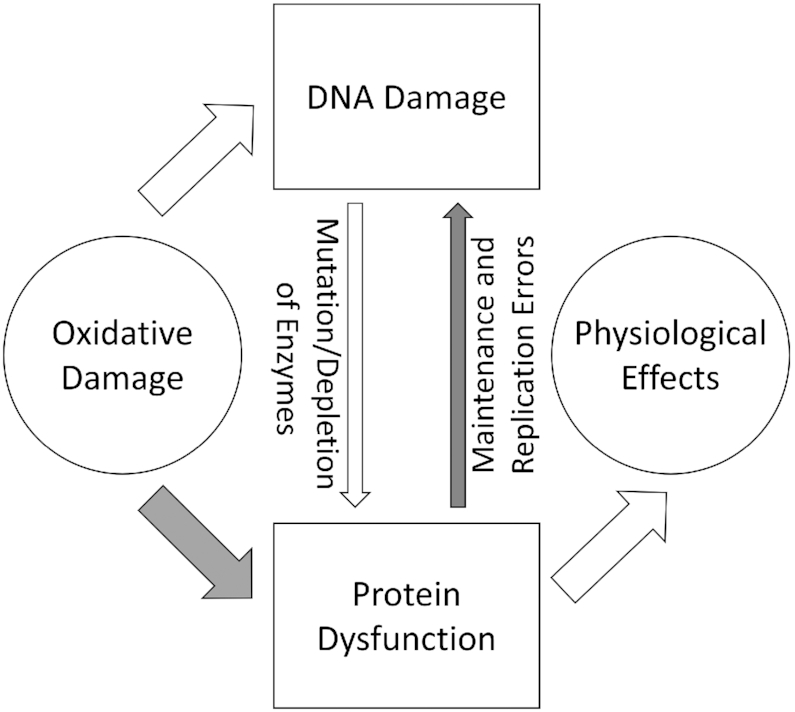
Cooperation for protein and DNA oxidative damage.

Oxidation damages both DNA and proteins, but proteins are far more sensitive to oxidation than DNA (44). Oxidation can disrupt protein-protein interactions (45), inhibit protein activity (46), or up-regulate protein expression level (47). The ROS imposes a threat particular to mitochondrial proteins, impairing metabolism and energy production. In nonalcoholic liver failure, more than 140 mitochondrial proteins are found to suffer from oxidative damage (48). We show here that oxidized Pol γ increases mtDNA mutations, which, in turn, could exacerbate mitochondrial oxidative stress that may further damage Pol γ, creating a cycle of oxidative stress (Figure 7). To alleviate oxidative damage, Pol γ, and perhaps many other mitochondrial proteins, should have evolved to be either more resistant to oxidation than nuclear polymerases or are turned over faster once oxidized. For example, Pol γA alone is more sensitive to oxidation, but becomes more resistant once complexed with the accessory subunit Pol γB (26).

Mass spectrometry is a reliable method for oxidative residue detection, among different experiments an oxidized amino acid can be identified with accuracy within two positions in protein sequence. The detection limit is due to difficulty in resolving adjacent oxidized amino acids. While oxidation induced protein backbone breakage can occur under extreme circumstances, side chain damages are more abundant and diverse at lower ROS. Interestingly, the oxidized amino acids we detected are not exclusively follow the reactive rate for amino acid oxidation measured individually (45), e.g. Tyr, Phe and Met have the highest rate constants as single amino acid, but were not abundant in oxidized Pol γ, suggesting the 3D structure or solvent accessibility play an important role in residue oxidation. Pol γ displays a few hotspots for oxidation via a variety of side chain oxidations. The most prominent one is located in the *exo* active site, which exhibits relatively large solvent access surface. Oxidation enhances the negative charge in the *exo* site thus reduces its affinity to DNA. Contrary to *exo* site, the *pol* site is buried in a deep cavity in the polymerase, with a narrow tunnel for transporting substrate nucleotide (27,32). This feature, conserved among Pol I family members, may be beneficial in discouraging dissociation of the bound nucleotide, and also in preserving the rudimentary activity of the polymerase under oxidation insult. This bias towards synthesis over error-correction may be embedded in the structure of the polymerases: during high fidelity DNA replication, the primer/template DNA is frequently transferred between Pol γ *pol* and *exo* sites that are separated by 35–40 Å for error correction. The shuttling between the two active sites is a slow step in DNA replication. If both *exo* and *pol* active sites were located in deep cavities, they may impose physical constraints that could limit primer strand transferring, reducing both replication rate and fidelity.

Pol γ is an adverse reaction target for antiviral reagents designed against HIV, especially the nucleoside analog inhibitors HIV RT (NRTIs). Low toxic NRTIs are either being discriminated against its incorporation in the *pol* or excised from the primer stand in *exo* site: the most toxic NRTIs, Zalcitabine (ddC) is least likely to be excised in Pol γ *exo*, whereas low toxic Emtricitabine (FTC) is excised from the primer DNA strand two orders of magnitude faster rate than ddC (49), emphasizing the important of the proofreading activity in reducing NRTIs drug toxicity. The mutator phenotype of oxidized Pol γ shows lower *exo* activity, potentially elevate antiviral drug toxicity. Conversely, NRTIs impose stress to mitochondria (50,51) and increase mtDNA replication errors. The observation shed lights on long-term usage of NRTIs resulted premature aging in patients.

## Supplementary Material

gkz1018_Supplemental_File
