# Supplementary material for: Oxidative damage diminishes mitochondrial DNA polymerase replication fidelity
Source: Nucleic Acids Res. 2019 Dec 4;48(2):817–29. doi: 10.1093/nar/gkz1018 (PMC6954441; doi:10.1093/nar/gkz1018)
Supplement: gkz1018_Supplemental_File [file gkz1018_supplemental_file.doc]

Supplementary materials


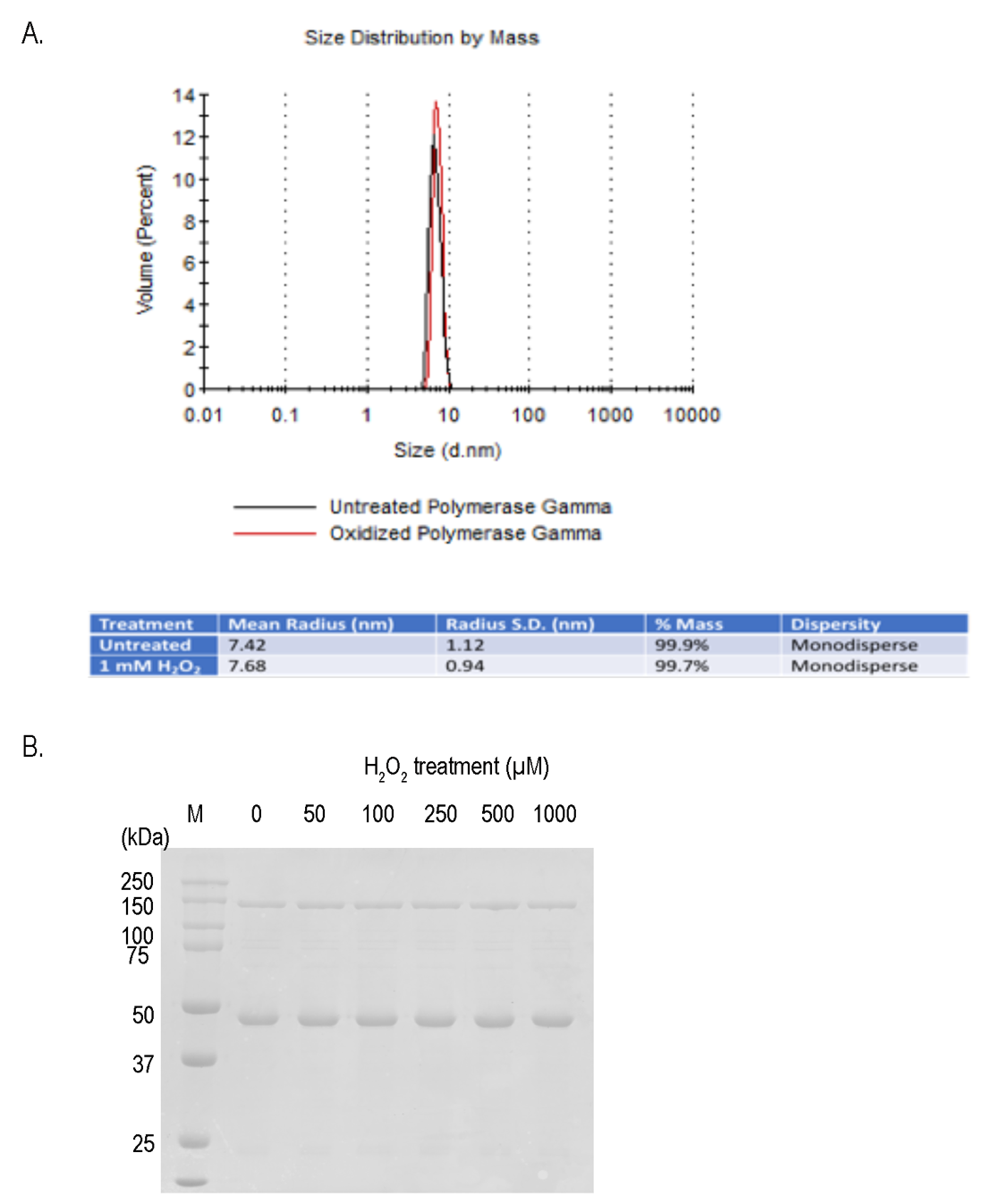


Fig. S1. Physical properties of oxidized Pol γ. A) Dynamic light scattering measurement for untreated and 1 mM H_2_O_2_ oxidized Pol γ. B) Untreated and H_2_O_2_ treated Pol γ resolved on an SDS gel.


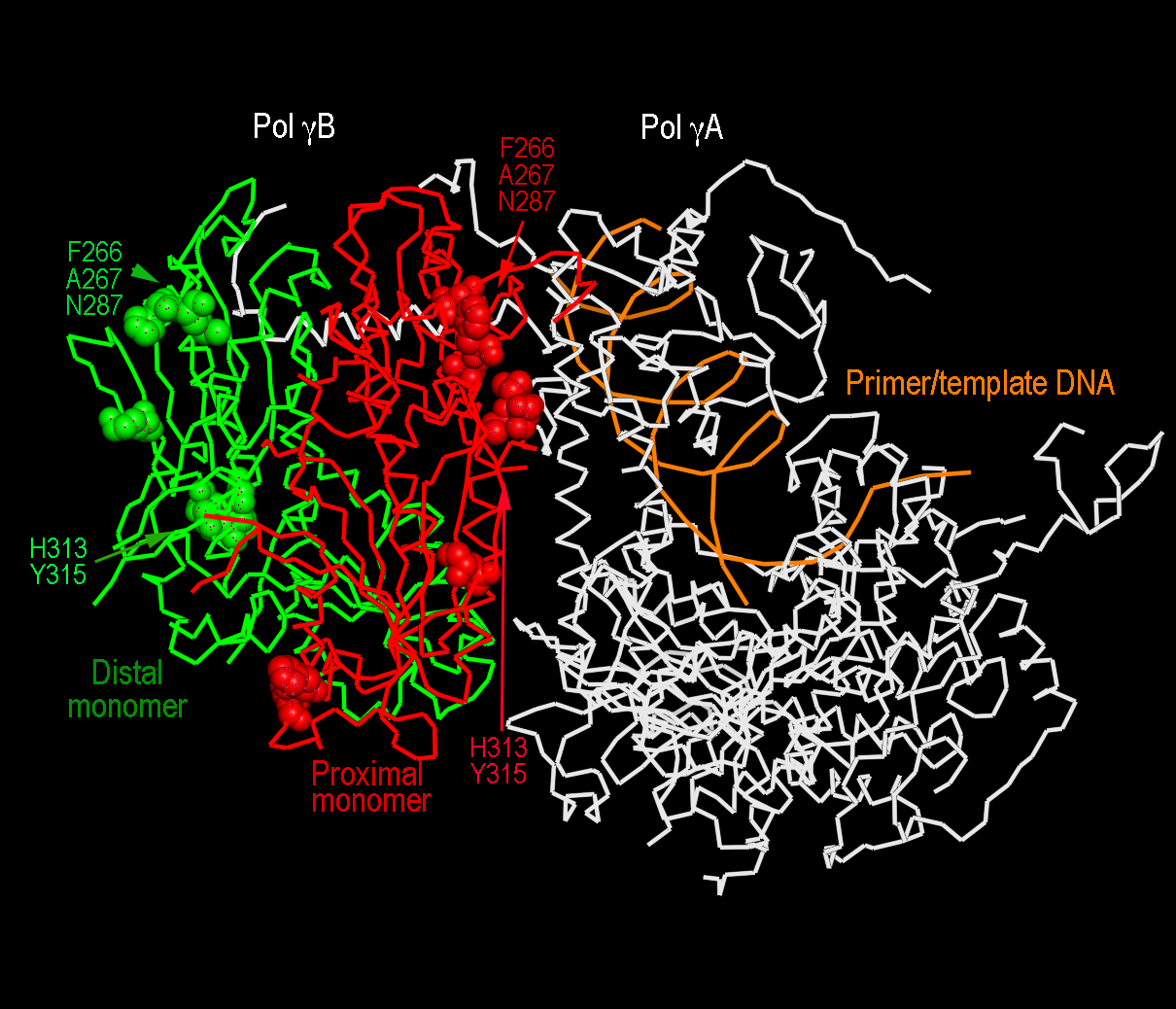


**Fig. S2. Locations of oxidized residues in Pol** γ **B.** The displayed holoenzyme consists of the distal Pol γ B monomer (colored in green), the proximal monomer (red) and the catalytic subunit Pol γ A (white). The detected oxidized residues in Pol γ B are shown on both proximal and distal monomers in spheres.

A.


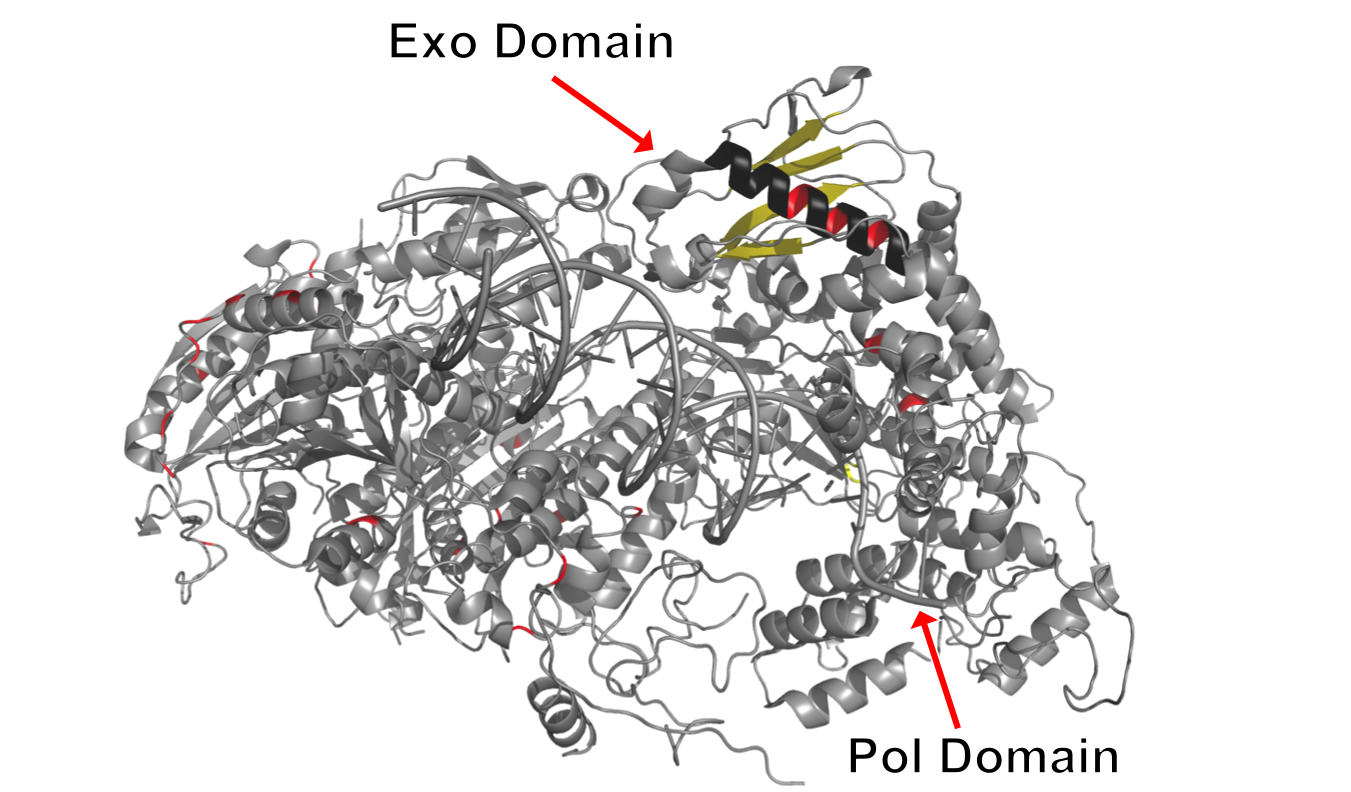


B.

**Figure S3.** **Oxidative modifications of Pol γ *exo* site.** A) Overall crystal structure (PDB code 4ztz) showing oxidatively modified residues around the exo active site, the unmodified residues are shown in black and oxidized in red). B) Closed views of the oxidized residues in exo site of untreated (Control) and oxidized Pol γ.
